# Supplementary material for: Differences in hospital admissions practices following self-harm and their influence on population-level comparisons of self-harm rates in South London: an observational study
Source: BMJ Open. 2019 Oct 17;9(10):e032906. doi: 10.1136/bmjopen-2019-032906 (PMC6803107; doi:10.1136/bmjopen-2019-032906)
Supplement: Supplementary data [file bmjopen-2019-032906supp001.pdf]

Self-harm and suicidality terms used in creation of dataset

'%self harm%'
'%self-harm%'
'% DSH %'
'% OD %'
‘'%overdose%'
'% cut%'
'%cutting%'
'%burning%'
'% burn%'
'% od.%'
'%o/d%'
'% dsh.%'
'%O.D%'
'%paracetamol%'
'%lacerat%'
'%immersion%'
%suicide attempt%'
'%attempting suicide%'
'% hang%'
'%noose%'
'%strangle%'
'%suffocate%'
'%helium%'
'%suicidal%'
'%suicidality%'
'%suicide%'
'%kill himself%'
'%kill herself%'
'%killing himself%'
'%killing herself %'
'%kill myself%'
'%harm himself%'
'%harm herself%'
'%harm myself%'
'%end his life%'
'%end her life%'
'%end my life%'
'%harm to self%'
'%end it all%'
