# Supplementary material for: Differences in hospital admissions practices following self-harm and their influence on population-level comparisons of self-harm rates in South London: an observational study
Source: BMJ Open. 2019 Oct 17;9(10):e032906. doi: 10.1136/bmjopen-2019-032906 (PMC6803107; doi:10.1136/bmjopen-2019-032906)

Residual standardised rate ratios (SRRs) for Emergency Department attendance and admission for self-harm for 725 Lower Super Output Areas (LSOAs) in study area

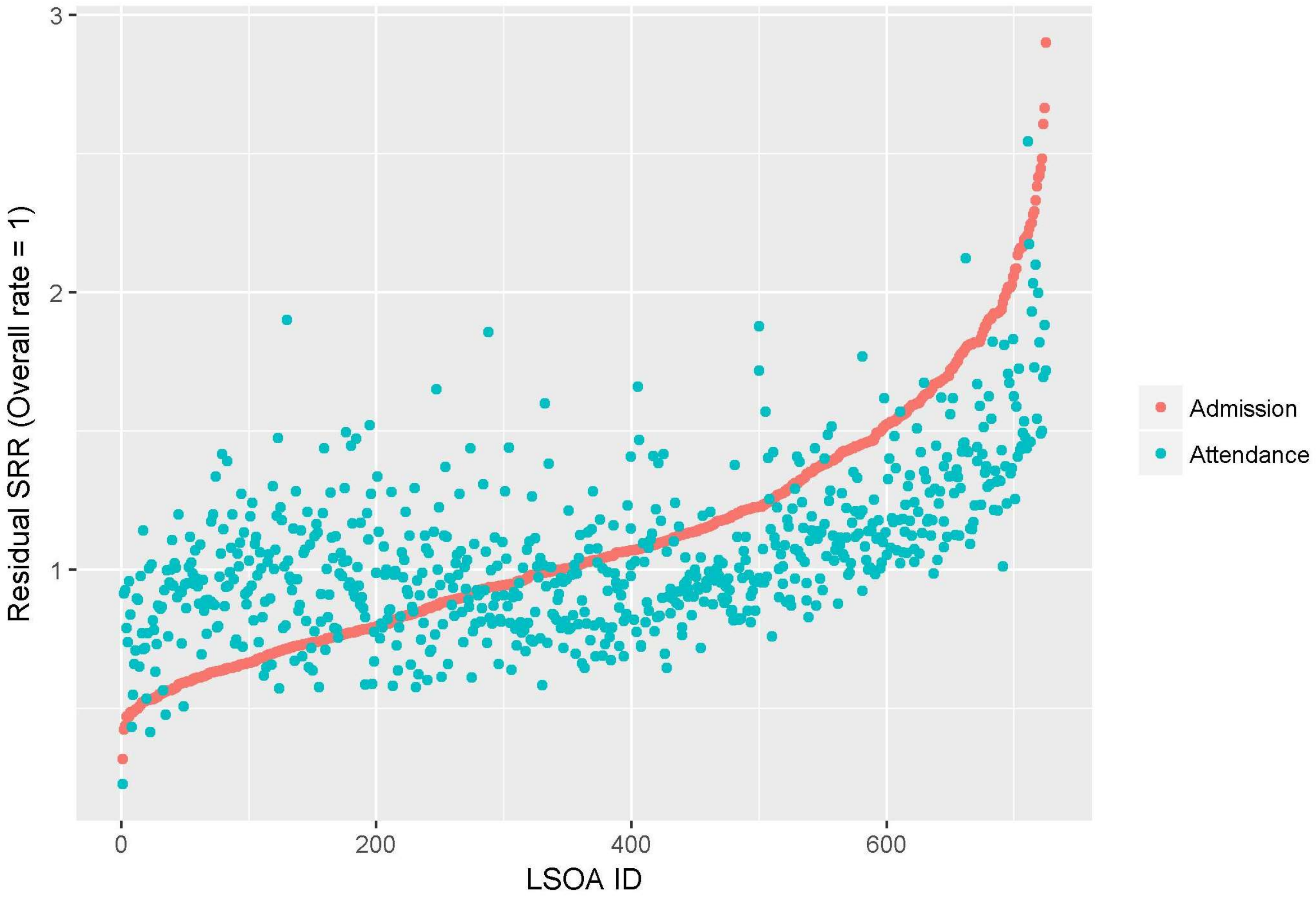

Supplement: Supplementary data [file bmjopen-2019-032906supp002.pdf]
