# Supplementary material for: Differences in hospital admissions practices following self-harm and their influence on population-level comparisons of self-harm rates in South London: an observational study
Source: BMJ Open. 2019 Oct 17;9(10):e032906. doi: 10.1136/bmjopen-2019-032906 (PMC6803107; doi:10.1136/bmjopen-2019-032906)
Supplement: Supplementary data [file bmjopen-2019-032906supp003.pdf]

a) First attendances b) First attendances adjusted for distance to hospital and c) First admissions by individuals aged 11+ for self-harm 2009-2016 by lower super output area, standardised for age and sex and adjusted for IMD

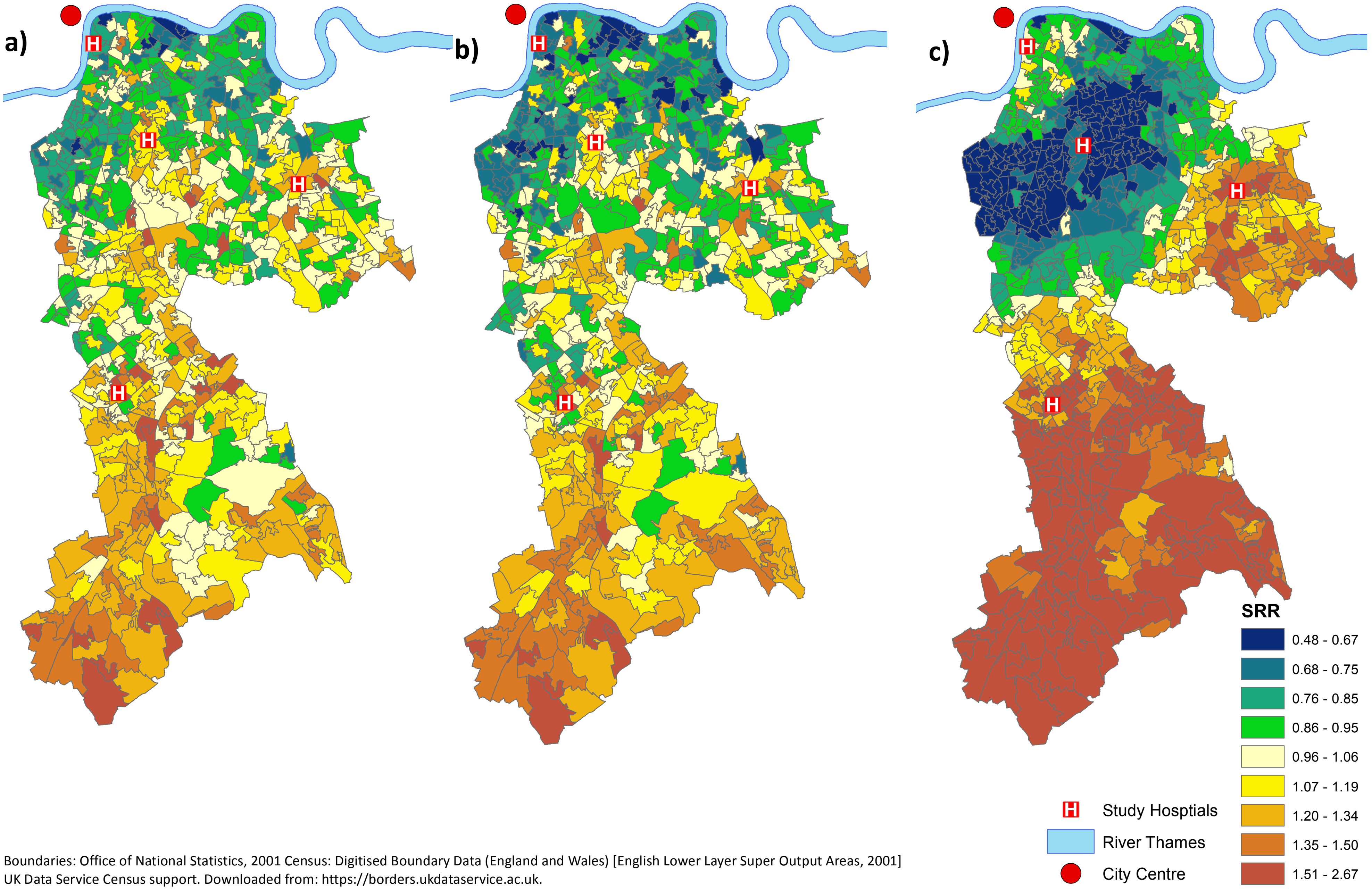

Boundaries: Office of National Statistics, 2001 Census: Digitised Boundary Data (England and Wales) [English Lower Layer Super Output Areas, 2001] UK Data Service Census support. Downloaded from: https://borders.ukdataservice.ac.uk.
